# Weight stigma in healthcare settings: the experience of Arab and Jewish bariatric surgery candidates in Israel

**DOI:** 10.1186/s13584-023-00587-4

**Published:** 2024-01-02

**Authors:** Yara Zahra-Zeitoun, Roni Elran-Barak, Rawan Salameh-Dakwar, Dvir Froylich, Gideon Sroka, Ahmed Assalia, Yael Latzer

**Affiliations:** 1 School of Public Health, University of Haifa, Haifa, Israel; 2 Carmel Medical Center, Haifa, Israel; 3 Bnai Zion Medical Center, Haifa, Israel; 4 Rambam Medical Center, Haifa, Israel

**Keywords:** Weight bias, Internalization, Bariatric surgery, Stigma, Obesity, Health settings

## Abstract

**Background:**

Weight-related stigma and discrimination are prevalent in our society with adverse biopsychosocial outcomes to people with obesity and morbid obesity. Studies suggest that weight bias in healthcare settings are quite prevalent, but there have been, as far as we know, lack of studies examining prevalence and correlates of weight bias experiences among bariatric surgery candidates in Israel. We aim to understand the nature and prevalence of weight stigma among bariatric surgery candidates. To identify differences between Jewish and Arab candidates. To examine the impact of weight stigma experiences on weight bias internalization (WBI).

**Methods:**

A cross-sectional study was performed among 117 adult bariatric surgery candidates from three hospitals in northern Israel (47.8% Jews, 82.4% females, average BMI 42.4 ± 5.2 Kg/meter^2^). Patients who agreed to participate completed a structured questionnaire on the same day that the bariatric surgery committee met. WBI was measured using a validated 10-item scale. Experiences of weight stigma were measured using items adapted from prior international studies.

**Results:**

About two thirds of the participants had at least one experience of weight stigma (teased, treated unfairly, or discriminated against because of their weight). As many as 75% of participants reported that weight served as a barrier to getting appropriate health care and as many as half of participants felt in the last year that a doctor judged them because of their weight. No significant differences were found between Arabs and Jews in the prevalence of weight stigma experiences and WBI. However, a trend towards more stigma experiences among Jews was noted. WBI was predicted by female gender and experiences of weight stigma, both in general and within healthcare settings.

**Conclusions:**

Weight stigma towards bariatric surgery candidates in Israel is quite prevalent, and specifically in healthcare settings. It is important to adopt policy actions and intervention programs to improve awareness to this phenomenon among the general public and specifically among healthcare providers, as many healthcare providers may be unaware of the adverse effect of weight stigma and of ways in which they are contributing to the problem. Future studies may validate our findings using larger sample size and longitudinal design.

## Background

Obesity rates have surged over the past 50 years, with global estimates of over 650 million people living with obesity (BMI > 30), accounting for more than 10% of the world's adult population [1]. In Israel, approximately 60% of adults aged 25–64 are classified as overweight (BMI > 25) or obese [2, 3], with higher prevalence observed among Arab residents compared to Jewish residents [2–5]. Bariatric surgery has emerged as an effective treatment for individuals with severe obesity who have not achieved weight loss through alternative interventions [1, 6]. Israel ranks fifth worldwide in terms of bariatric procedures performed per resident [7], and the rate of bariatric surgery among the Arab population in Israel has seen a notable increase from 16.5% of all surgeries in 2014 to 26% in 2018, possibly influenced by heightened health awareness and concerns about physical appearance within the Israeli Arab community [8]. It is worth noting that in 2017, approximately 10,000 bariatric surgeries were conducted in Israel, however the number of surgeries has seen a consistently decline since then [9].

Weight stigma is a social devaluation of individuals due to their body weight and is one of the most common forms of discrimination in modern society [10]. Weight-based stereotypes refer to false or misleading generalizations about individuals with obesity (e.g., that they are lazy, lacking in will power and discipline, unmotivated, or uncompliant). Individuals with obesity are stigmatized and discriminated against in nearly all major life domains such as employment, healthcare, and education, at great cost to their well-being [11]. Weight stigma has serious psychological, physiological, and social consequences [12–19]. For example, it can lead to social avoidance, lower self-esteem, damaged psychological well-being, and impaired eating patterns, which can ultimately harm weight loss efforts [20]. Despite the understanding that weight stigma is common, prevalent, and has adverse consequences, data about weight stigma in Israel, and specifically in healthcare settings, are lacking.

Studies suggest that physicians and health providers are one of the most frequent sources of weight bias [20]. It has been found that about half of women with obesity and overweight report receiving inappropriate comments from medical doctors and nurses about their weight [21]. Experiences of weight stigma from other health professionals, including dietitians and mental health professionals, are also quite prevalent [20]. Stigma in healthcare settings may deter individuals from seeking medical treatments due to fear of judgment, leading to negative health outcomes and reduced access to preventive care [22]. One prior survey, which was conducted in Israel among social-media respondents with overweigh (BMI > 25), reports that about half of respondents have received suboptimal treatment related to excess weight (e.g., insulting and judgment approach), which may impact avoidance of medical treatment, but data among clinical samples are unavailable [23].

Weight bias internalization (WBI) refers to internalizing negative weight stereotypes, leading to self-disparagement [20]. Higher BMIs and previous experiences of weight stigma, including unfair treatment, teasing, and discrimination, are commonly associated WBI [24], which can lead to negative biopsychosocial outcomes [25]. Importantly, WBI is associated with weight gain, eating disturbances, depression, anxiety, body dissatisfaction, low self-esteem [15, 16], and increased risk of suicidal thoughts and behaviors [19]. WBI is particularly problematic among bariatric patients (BMI > 40 or BMI ≥ 35 with physical comorbidities) given that it predicts worse dietary adherence, even after weight is lost [26]. Among bariatric patients, WBI is significantly associated with greater eating-disorder psychopathology, depression [27], and lower perceived mental quality of life following surgery [28].

Given the high obesity prevalence among Arab population in Israel [3], exploring weight stigma among Jewish and Arab Israeli residents is important. Studies on weight bias among ethnic/racial groups suggest that Caucasians may be more susceptible to weight bias than other ethnic/racial groups [29]. Research in the U.S [30] reveals lower WBI among African Americans with obesity [31], particularly those with a stronger ethnic identity [32]. This finding may suggest that ethnicity serves as a protective factor against the internalization of the “thin ideal” [33]. However, conflicting evidence suggests that ethnic minorities in the U.S [34] (including African Americans [35]) report more weight-based teasing and discrimination relative to their Caucasian counterparts. Examining weight bias among Arabs in Israel is crucial, as most research has focused on ethnic minorities in the U.S. and not in other parts of the world.

Limited literature exists on weight stigma prevalence in Israel, particularly within different ethnic/racial groups, and there is a lack of data regarding its correlates [23]. One study that examined attitudes and practices among Israeli primary care physicians suggested that up to one third of physicians held disparaging attitudes toward overweight patients (e.g., that overweight people are lazier than normal weight people) [36]. An additional study that used qualitative methods demonstrated negative feelings and behaviors among Israeli dietitians toward their patients with obesity [37]. These studies provide important information about weight-based stigma, yet data about weight bias (including data about weight-bias experiences and WBI) among different ethnic groups in Israel, and specifically among Arab vs. Jewish bariatric surgery candidates, are still missing.

The aims of this study were therefore:

- To examine the prevalence of experiences with weight stigma and weight bias in general and in healthcare settings among Israeli samples of bariatric surgery candidates.
- To examine differences between Arabs and Jews in the prevalence of experiences with weight stigma and weight bias in general and in healthcare settings.
- To examine differences between Arabs and Jews in WBI.
- To examine if experiences of weight stigma and weight bias can cross-sectionally predict WBI.

## Methods

### Procedure

This is a cross-sectional study. The study population included a convenience sample of candidates attending the bariatric committee meeting at three hospitals in northern Israel between 2019 and 2022: Rambam Health Care Campus (*n* = 39, from Sep 2019 until Feb 2020), Bnai Zion Medical Center (*n* = 38, from Nov 2021 until Sep 2022) and Carmel Medical Center (*n* = 40, from Nov 2021 until Dec 22). On the day of the bariatric surgery committee meeting, an explanation about the research was given to the surgery candidates. Participants were assured of full confidentiality as all questioners were anonymous. A research assistant was present at the hospital on the days of the committee to distribute the questionnaires to potential participants. It is worth noting that whenever the research assistant was available to be present, a high response rate of approximately 90% was achieved. After obtaining informed consent, participants were provided with paper and pencil anonymous questionnaires (self-completion). The inclusion criteria for participants were adults aged 18 years or older, with a BMI greater than 35, and the ability to read and write.

### Study population

The study included 117 adults over 18 years of age, comprising 61 Arabs (52%) and 56 Jews (48%). Among the Arab group, 46% self-reported they were Muslims, 8% self-reported they were Christians, and 7% self-reported they were Druze.

### Measures

All items were taken from prior studies as explained below. If no validated Hebrew/Arabic version existed, we used the back-translation method to translate the questionnaire.

### The experience of weight stigma

Was evaluated using yes/no items from prior national studies conducted in the U.S. [38–41] in which participants were asked three questions: ״Have you ever been (1) teased, (2) treated unfairly, or (3) discriminated against because of your body weight?” 

### Weight stigma in healthcare settings

Prior experience of weight stigma in health care was assessed the following item ‘‘In the last 12 months, did you ever feel that a doctor judged you because of your weight?”. The 3-option scale (never, sometimes, or often) was reduced to two options (never or at least once) as was done in prior studies [22]. Participants were also asked the following question: “Has your weight been a barrier to getting appropriate health care? (yes/no)” [42–44]. In addition, participants were asked to follow the following instructions: “Please select any of the following that you have personally experienced in your health care: (1) being treated disrespectfully by health care providers (e.g., doctors, nurses) because of your weight, (2) feeling embarrassed or ashamed about being weighed at the doctor’s office (3) negative attitudes or remarks of health care providers (e.g., doctors, nurses) about your weight (4) having a doctor recommend a diet, even if you did not come in to discuss weight loss (5) Not being able to find medical equipment (e.g. blood pressure cuffs, patient gowns) in a size that works for you or fits you properly (6) having a doctor tell you to lose weight, but not providing you with weight loss treatment options or advice on how to get help for weight loss. (7) A doctor blaming unrelated physical problems on your weight. A sum of these elements was calculated (min = 0, max = 9) to signify how strongly participants experienced weight stigma in a healthcare setting.

### Weight bias internalization scale (WBI scale)

The original WBI scale is an 11-item instrument with excellent psychometric properties and high construct validity [45]. However, recent research conducted specifically on patients presenting for bariatric surgery suggested removing the first item of the scale (“As on overweight person, I feel that I am just as competent as anyone”) to improve internal consistency, yielding a 10-item measure (Cronbach’s *α* = 0.90) [46], which we have used in the current study. Participants rated their agreement with each statement on a 7-point Likert scale ranging from 1 (strongly disagree) to 7 (strongly agree). (1) I am less attractive than most other people because of my weight, (2) I feel anxious about being overweight because of what people might think of me, (3) I wish I could drastically change my weight, (4) Whenever I think a lot about being overweight, I feel depressed, (5) I hate myself for being overweight, (6) My weight is a major way that I judge my value as a person, (7) I don’t feel that I deserve to have a really fulfilling social life, as long as I’m overweight, (8) I am OK being the weight that I am, (9) Because I’m overweight, I don’t feel like my true self, (10) Because of my weight, I don’t understand how anyone attractive would want to date me [45]. Cronbach’s *α* in this study was 0.708.

### Statistical analysis

The data were analyzed using SAS version 9.4 and *p* < 0.05 was considered significant. Categorical data were reported as the number (%) and were compared using the chi–square test. Continuous variables were reported as mean ± SD, and between-group comparisons were performed using a *t*-test or the two-samples Wilcoxon test. In order to examine the associations between experiences of weight stigma and WBI, we initially examined the univariate associations between participants characteristics, their weight stigma experiences, and WBI. Subsequently, in the multivariate analysis that predicts WBI, we included only those independent variables that showed significant associations with WBI in the univariate analysis. This approach helped us reduce the number of independent variables to five in the final linear regression model.

## Results

A summary of participant characteristics appears in Table 1. Sixty-one participants were Arabs, and 56 were Jews. Mean age was 38.9 years of age (SD = 11.9). Mean age of Jewish bariatric candidates was 41.4 (SD = 13.2), significantly (*p* = 0.027) higher than that for Arab bariatric candidates, 36.5 years of age (SD = 10). Participants consisted of 89 women and 19 men (18%). Of the Arab candidates, 91.1% were women, whereas 73.1% of the Jewish candidates were women (*p* = 0.014). Of the Arab participants, 63.6% lived alone, compared to 47.3% of the Jewish participants (*p* = 0.083). About 69.6% of all the participants had children, with no significant differences between Arabs and Jews (*p* = 0.764). Out of all the participants, 16.4% had 12 years of education, and 83.6% had a higher education (college/university), with no differences between Arabs and Jews (*p* = 0.856). Among Arabs, 38.3% were employed, and among Jews, 23.1% were employed (*p* = 0.082). Income was significantly higher among Jews than among Arabs (*p* = 0.045). Mean BMI among all participants was 42.4 (SD = 5.2), with no significant difference between Arabs and Jews (*p* = 0.888).

**Table 1:** Characteristics of participants

	Arabs n = 61		Jews n = 56		Overall n = 117		*p* value
*Age*							
Mean ± SD	10.0 ± 36.5		13.2 ± 41.4		11.9 ± 38.9		0.027
*Gender*							
%Female	91.1%	n = 51	73.1%	n = 38	82.4%	n = 89	0.014
*Family status*							
%Living alone	63.6%	n = 38	47.3%	n = 26	55.7%	n = 64	0.083
*Have kids*							
%Yes	68.3%	n = 41	70.9%	n = 39	69.6%	n = 80	0.764
*Education*							
% < 12 years	15.1%	n = 8	16.4%	n = 9	15.7%	n = 17	0.856
*Work status*							
%Working	38.3%	n = 23	23.1%	n = 12	31.3%	n = 35	0.082
Income ≤ 6,000 NIS	48.1%	n = 25	25.0%	n = 12	31.6%	n = 37	**0.045**
*Income (per household)*							
6,000 < Income ≤ 12,000 NIS	26.9%	n = 14	37.5%	n = 18	27.4%	n = 32	
Income > 12,000 NIS	25.0%	n = 13	37.5%	n = 18	26.5%	n = 31	
*BMI*							
Mean ± SD				42.5 ± 5.0	42.4 ± 5.4	42.4 ± 5.2	0.888

Significant *p* values appear in bold

Table 2 shows that no significant differences in any of the three items of weight stigma experiences were found between Arabs and Jews. Of all of the bariatric candidates, 65.8% reported at least one stigmatizing experience based on weight (Teased based on weight, Treated unfairly based on weight, Discriminated against based on weight). Table 3 demonstrates that out of all the participants, 50.0% of the Arab participants and 55.4% of the Jewish participants reported being judged by a medical doctor because of their weight. Almost three quarters of all participants, both Arabs and Jews, thought their weight served as a barrier to receiving proper medical treatment. No significant differences were found between Arabs and Jews. However, in almost all kinds of experiences, the bariatric candidates who were Jewish reported more weight stigma in healthcare settings than did those who were Arab, yet the figures were not statistically significant. Mean scores of internalized weight stigma also didn’t differ between Arabs and Jews (4.5 ± 1.3 and 4.1 ± 1.5, respectively).

**Table 2:** Weight bias experiences (general)

	Arabs n = 61	Jews n = 56	p value
Teased based on weight	57.4%, n = 35	55.4%, n = 31	0.825
Treated unfairly based on weight	52.5%, n = 32	42.9%, n = 24	0.299
Discriminated against based on weight	49.2%, n = 30	32.1%, n = 18	0.061
At least one stigmatizing experience	**64.3%, n = 36**	**67.2%, n = 41**	**0.733**

Signtificant values appears in bold

**Table 3:** Experiences of weight stigma in healthcare settings and weight bias internalization (WBI) scores

	Arabs N = 61	Jews N = 56	*P value*
In the last 12 months, did you ever feel that a doctor judged you because of your weight?	50.0%, n = 30	55.4%, n = 31	0.564
Has your weight been a barrier to getting appropriate health care?	75.4%, n = 46	75.0%, n = 42	0.959
*Please select any of the following that you have personally experienced in your health care*			
1. Being treated disrespectfully by health care providers (e.g., doctors, nurses) because of your weight,	11.5%, n = 7	14.3%, n = 8	0.649
2. Feeling embarrassed or ashamed about being weighed at the doctor’s office	54.1%, n = 33	42.9%, n = 24	0.224
3. Negative attitudes or remarks of health care providers (e.g., doctors, nurses) about your weight	13.1%, n = 8	19.6%, n = 11	0.338
4. having a doctor recommend a diet, even if you did not come in to discuss weight loss	50.8%, n = 31	66.1%, n = 37	0.065
5. Not being able to find medical equipment (e.g. blood pressure cuffs, patient gowns) in a size that works for you or fits you properly	9.8%, n = 6	10.7%, n = 6	0.875
6. having a doctor tell you to lose weight, but not providing you with weight loss treatment options or advice on how to get help for weight loss	19.7%, n = 12	23.2%, n = 13	0.640
7. A doctor blaming unrelated physical problems on your weight	22.9%, n = 14	37.5%, n = 21	0.086
Sum of 9 experiences (Mean ± SD, range: 0–9)	2.5 ± 2	3.0 ± 2.1	0.313
WBI – mean score	4.5 ± 1.3	4.1 ± 1.5	0.065

Table 4 presents a linear regression model predicating WBI. The number of observations used for the model was 101, as 16 observations were missing values. The adjusted R-squared for the model was 0.293. Age, BMI, education, and ethnicity (Arab compared to Jewish) were not found to predict WBI. However, female gender and the sum of the three general experiences of weight stigma (teasing, discrimination, and unfair treatment based on weight) significantly predicted WBI (*p* < 0.001). Concurrently, the sum of the 9 items of weight stigma experiences also significantly predicted WBI (*p* < 0.001).

**Table 4:** Univariate and Multivariate Associations between participants characteristics and weight bias internalization (WBI)

Variable	Univariate Associations with WBI		Multivariate Associations with WBI	
	Pearson Correlation	P-value	StandardizedBeta	P-value
Age	**-.246**	**.008**	-.007	.527
BMI	.073	.449	–	–
Female Gender	**.227**	**.018**	**.697**	**.034**
Education (< 12 years)	**-.195**	**.043**	-.167	.530
Arabs compared to Jews	.171	.065	–	–
Sum of three items of general weight stigma experiences	**.422**	**.000**	**.367**	**.001**
Sum of 9 items of weight stigma in healthcare settings	**.443**	**.000**	**.181**	**.004**

Signtificant values appears in bold

## Discussion

This study was designed to examine the prevalence of weight stigma among bariatric candidates in Israel. Several important findings emerged from our analyses. First, the results provide evidence that experiences of weight bias are common among bariatric candidates, both in healthcare settings and in other circumstances. As many as about three quarters of participants reported that their weight was a barrier to getting appropriate health care, and about half of participants feel that in the last year, a doctor judged them because of their weight. Second, there were no significant differences between Arabs and Jews in terms of weight stigma experiences and internalization. However, it is worth noting that there was a noticeable trend towards more weight stigma experiences among Arabs, although this trend did not reach statistical significance. Third, female gender and experiences of stigma significantly predicted WBI. These findings highlight the notion that weight stigma is quite prevalent among bariatric candidates in Israel.

Approximately two-thirds of our bariatric candidates reported experiencing weight stigma, such as teasing, unfair treatment, or discrimination based on their weight at any point in their lives. These rates are relatively high compared to previous studies. A similar study conducted in the U.S. among patients with obesity found that about half of the participants reported some form of weight stigma experience [40]. These differences could be attributed to the fact that our sample consisted mostly of women (82.4%, *n* = 89), with higher average BMI, both of which are linked with a higher prevalence of weight stigma experiences [47]. The most frequent stigmatizing experience was being teased on the basis of weight, in both the Arab and Jewish groups, yielding a prevalence of 56.4%, a finding that is consistent with findings from previous, larger population studies, with a prevalence of 47% (*N* = 3821) [40] and 45.4% (N = 3504) [41].

In terms of weight stigma in healthcare settings, this study found that three quarters of participants experienced weight as a barrier to receiving adequate medical treatment. Additionally, over half of the participants reported feeling judged by a doctor within the past year. These findings are consistent with prior research indicating that doctors are a common source of stigma [48]. A Swedish survey with a community-based sample (*N* = 2788) also reported a comparable prevalence of 40% of individuals with class II/III obesity experiencing weight-related judgment from doctors [47].

In our study, the most frequently reported stigmatizing experience was "a doctor recommending a diet even if you did not come in to discuss weight loss," reported by 58% of the sample. This prevalence was higher than a previous study, where it was 34% [49]. The difference may be due to the lower average BMI in the prior study (around 35 kg/m [2]) compared to our study (42 kg/^m2^), as higher BMI is associated with more stigmatizing experiences [49]. The second most endorsed item was "feeling ashamed while physicians weighed me," reported by 48.7% of participants. This experience is linked to body-related shame, healthcare-related stress, and avoidance of healthcare services [50]. An encouraging finding is that only a small percentage of participants reported equipment unavailability (9.8–10.7%), indicating that most clinics in Israel are well-equipped for patients with obesity, consistent prior findings [23, 51].

A nonsignificant trend was found when examining differences between Arabs and Jews in prevalence of weight stigma experiences, suggesting that weight stigma experiences may be slightly more common among Jews relative to Arabs. Mixed and inconsistent results have been found in previous studies evaluating the association between weight stigma and race/ethnicity. For example, one study conducted in the U.S. suggested that African American individuals, particularly women, were less likely to have negative attitudes toward people with obesity, compared to Caucasians [52]. However, contrasting findings have been found in other studies in the U.S., suggesting a higher prevalence of weight stigma among ethnic minorities [34, 35]. During the past decades, Arab society in Israel has undergone major changes in lifestyle [53]. Although plumpness in the Arab culture has traditionally been seen as a symbol of femininity, motherhood, sexuality, and fertility [54], the internalization of Western beauty ideals and the idealization of thinness have grown stronger, even among people who traditionally and culturally never previously held these values [55, 56]. Additionally, it has been suggested in previous studies that ethnic minorities reject the standards and ideals of the majority group, such as the thin ideal model, for example—to protect their sense of uniqueness [57]. Nevertheless, there are currently high rates of body dissatisfaction and disordered eating among Arab youth in Israel [58, 59], and therefore, it is not surprising that our findings suggest high rates of weight stigma experiences and internalization among both Jewish and Arab participants.

To the best of our knowledge, the current study is the first one in Israel to investigate the prevalence of weight stigma among Arab and Jewish bariatric surgery candidates. The results of this study provide useful and important information about the prevalence of weight stigma in Israel and the associated internalization of such negative stereotypes. That said, this study had several limitations. First, our study utilized a relatively small sample, which may have limited the statistical power to detect small groups differences. This sample size could potentially be insufficient to accurately identify small differences among the studied groups (e.g., differences in weight stigma encounters). Therefore, it is important to interpret the findings with caution and acknowledge trends in nonsignificant differences. Second, this was a convenient and unrepresentative sample. The fact that all the participants were selected from three medical centers in the north of the country means that this group does not represent the entire population of individuals with obesity in Israel. In addition, participants were required to possess reading and writing abilities, which may limit the inclusion of individuals with low literacy levels, potentially limiting the generalizability of the findings to the broader population. Third, the data in this study were self-reported and therefore subject to reporting bias and respondent bias. Another potential bias to consider is the influence of answering a survey about weight stigma experiences immediately before reporting WBI. Participants' heightened awareness of negative stereotypes and biases associated with weight during the survey could contribute to an increased internalization of those stigmas. Moreover, the cross-sectional nature of this study makes it difficult to determine a causal link between experiences of weight stigma and the internalization of this stigma. Lastly, BMI was calculated according to self-reported weight and height.

## Conclusion

Findings emphasize the need to increase public and professional awareness about weight stigma and develop intervention programs aimed at reducing it. Healthcare providers should be mindful of their attitudes towards individuals with obesity in order to create a supportive and non-stigmatizing healthcare environment. Moreover, these findings have important implications for health policy. The high rates of weight stigma reported among both Arab and Jewish bariatric surgery candidates in Israel highlight the necessity of allocating funding to weight stigma prevention efforts, such as training healthcare providers. Additionally, since a significant proportion of participants indicated that weight serves as a barrier to receiving appropriate healthcare, policy actions should focus on raising awareness about weight stigma among the general public and healthcare providers. It is crucial to develop and implement intervention programs addressing weight stigma in healthcare settings, as many providers may be unaware of the effects of weight stigma and their contribution to the problem.

## Data Availability

The datasets used and analyzed during the current study are available from the corresponding author on reasonable request.

## Abbreviations

BMI: Body mass index
WBI: Weight bias internalization
